# Methylation Status of Gene Bodies of Selected microRNA Genes Associated with Neoplastic Transformation in Equine Sarcoids

**DOI:** 10.3390/cells11121917

**Published:** 2022-06-14

**Authors:** Klaudia Pawlina-Tyszko, Ewelina Semik-Gurgul, Tomasz Ząbek, Maciej Witkowski

**Affiliations:** 1 Department of Animal Molecular Biology, National Research Institute of Animal Production, Krakowska 1 St., 32-083 Balice, Poland; ewelina.semik@iz.edu.pl (E.S.-G.); tomasz.zabek@iz.edu.pl (T.Z.); 2 Institute of Veterinary Medicine, University Centre of Veterinary Medicine JU-AU, Al. Mickiewicza 24/28, 30-059 Cracow, Poland; mawitkow@gmail.com; 3 Horse Clinic Służewiec, Puławska 266 St., 02-684 Warsaw, Poland

**Keywords:** equine sarcoid, skin tumor, DNA methylation, microRNA, gene body

## Abstract

Horses are of great importance in recreation, livestock production, as working animals in poorly developed countries, and for equine-assisted therapy. Equine sarcoids belong to the most commonly diagnosed tumors in this species. They may cause discomfort, pain, and can lead to the permanent impairment of motor function. The molecular bases of their formation are still under investigation. Our previous studies revealed altered microRNA (miRNA) expression and DNA methylation levels in sarcoid tumors. Abnormal patterns of methylation may be responsible for changes in gene expression levels, including microRNAs. Recently, the DNA methylation of gene bodies has also been shown to have an impact on gene expression. Thus, the aim of the study was to investigate the methylation pattern of gene bodies of chosen miRNAs identified in sarcoid tissue (miR-101, miR-10b, miR-200a, and miR-338-3p), which have also been established to play roles in neoplastic transformation. To this end, we applied qRT-PCR, Bisulfite Sequencing PCR (BSP), and Mquant methods. As a result, we identified the statistically significant downregulation of pri-mir-101-1, pri-mir-10b, and pri-mir-200a in the sarcoid samples in comparison to the control. The DNA methylation analysis revealed their hypermethylation. This suggests that DNA methylation may be one mechanism responsible for the downregulation of theses miRNAs. However, the identified differences in the methylation levels are not very high, which implies that other mechanisms may also underlie the downregulation of the expression of these miRNAs in equine sarcoids. For the first time, the results obtained shed light on microRNA expression regulation by gene body methylation in equine sarcoids and provide bases for further deeper studies on other mechanisms influencing the miRNA repertoire.

## 1. Introduction

Sarcoids are considered to be among the most commonly diagnosed tumors in equids. It is estimated that they may constitute between 12% and 67% of all equine tumors, and as much as 70% of all equine skin tumors [1,2,3]. The phenotype of this type of cancer embraces not only flat hairless lesions, but also fibroblastic nodules and massive tumors. Although they are not lethal, more aggressive forms may cause significant discomfort, pain, and hinder the use of the animal as well as lead to the permanent impairment of motor function [4], while protracted, unsuccessful treatment may end in euthanasia. As a consequence, sarcoids reduce an animal’s value and cause considerable economic losses for the owners.

Bovine papillomavirus (BPV) infection is widely accepted as the causative agent; however, the mechanism has not been fully characterized [5,6]. Thus, the knowledge on this type of cancer is still not exhaustive, which, along with the variety of sarcoid types, make successful treatment difficult and usually bears significant practical and financial implications [7].

One of the sequence types which may serve as a useful biomarker for prognosis and diagnosis as well as treatment targets, is microRNAs. They are ~22 nucleotides long, belong to the class of small non-coding RNAs, and do not code for proteins. However, they have the ability to influence gene expression levels owing to mRNA binding and blocking their further processing [8,9]. The vast network of miRNA interactions makes them participate in and regulate a plethora of crucial biological processes. Disrupted miRNA expression patterns have been identified in many diseases, including cancer. These sequences were associated with tumor progression [10], metastasis [10,11], and drug resistance [11,12]; thus, many of them function as oncogenes and tumor suppressor genes.

Our previous study also revealed altered miRNA expression levels in sarcoid tumors [13]. We characterized the expression signature of these miRNAs and identified over 100 miRNAs being differentially expressed in sarcoid tissue in comparison to healthy controls by applying next-generation sequencing [13]. Many of them have been shown to participate in neoplastic transformation in humans [11,12].

Gene expression, including that of microRNAs, is associated with specific DNA methylation patterns. Alterations leading to abnormal patterns embrace the hyper- and hypomethylation of promoter regions, which is a well-known mechanism in cancer which may lead to further progression [14,15]. However, recently, the DNA methylation of gene bodies and intergenic regions has also been gaining relevance due to its impact on gene expression [16,17,18].

The number of known microRNA genes subjected to methylation changes is still being broadened. For example, the methylation-driven silencing of the miR-34 family (miR-34a, miR-34b, and miR-34c) was identified in different types of human neoplasms, namely melanoma, oral, esophagus, stomach, colon, pancreas, breast, lung, and kidney cancers [19]. On the other hand, the hypermethylation of gene promoters of the miR-124 family was established in colon cancer, acute lymphoblastic leukemia (ALL), and cervical cancer [19]. Nevertheless, knowledge about epigenetic changes characterizing different cancer types is still insufficient and mainly limited to human data. This may be exemplified by equine sarcoids, for which only a few studies characterizing overall [20] and gene-specific [21,22,23] methylation patterns have been published.

The comprehensive characterization of DNA methylation changes is not only of value from a scientific point of view, but also has great potential to be applied in diagnostics and prognostics. The disrupted methylation of the promoter regions of genes and microRNAs may be characteristic for a specific cancer type [24], and may also be associated with the invasiveness of a tumor [25,26]. Moreover, knowledge of epigenetic modifications could also be used in treatment and could constitute a basis for new therapeutical approaches [24], e.g., embracing the induced expression of silenced miRNA genes. Thus, a search for epigenetic markers suitable for early diagnosis and the prediction of the effectiveness of a treatment for human and animal tumors has been of topical research interest.

Having taken into consideration the importance of miRNA and DNA methylation alterations in neoplastic transformation, we hypothesized that aberrant gene body methylation patterns may be one mechanism responsible for disrupted expression levels of miRNAs in sarcoid tissue samples. Thus, the aim of the study was to reveal the methylation patterns of chosen microRNA genes which were identified as aberrantly expressed in sarcoid tissue samples in our previous research (miR-101, miR-10b, miR-200a, and miR-338-3p), and play vital roles in cancer development [13]. To the best of our knowledge, this is the first study to shed light on the methylation status of gene bodies of microRNA genes in equine sarcoids. It will not only broaden our knowledge on this type of tumor, but also provide information for the growing area of research that investigates the roles and influence of gene body methylation.

## 2. Material and Methods

### 2.1. Research Material

Tissue samples from 12 equine sarcoids were collected in Horse Clinic Służewiec in Warsaw, Poland. The samples were provided from equids treated for equine sarcoid disease in the clinic. Since the samples constituted a byproduct of the performed surgical procedures, part 1, division 1.2, paragraph 1 of “Legislation for the protection of animals used for scientific or educational purposes” of Poland, stating that no Ethics Committee approval is needed when providing veterinary services, was applied. Moreover, all efforts were made to minimize the suffering of the animals. Twelve healthy skin samples were collected at a slaughterhouse, so no ethics committee approval was needed.

Immediately after excision, the samples were immersed in RNAlater Stabilization Solution (Ambion; Thermo Fisher Scientific, Waltham, MA, USA) and stored at −20 °C. DNA extraction was performed using the Sherlock AX (A&A Biotechnology, Gdynia, Poland) kit, while total RNA was isolated using the Direct-zol RNA kit (Zymo Research, Irvine, CA, USA), following the manufacturers’ protocols. The obtained DNA and RNA isolates were quantified on a NanoDrop spectrophotometer (Thermo Fisher Scientific, Waltham, MA, USA), and RNA quality control was carried out with the use of a 2200 TapeStation instrument (Agilent Technologies, Santa Clara, CA, USA). Genomic DNA was treated with sodium bisulfite using the EZ DNA Methylation-Gold Kit (Zymo Research, Irvine, CA, USA) according to the manufacturer’s protocol.

### 2.2. BPV and microRNA Gene Expression

The obtained control skin RNA isolates were tested for the expression of BPV genes to exclude ongoing BPV infection. For this purpose, 400 ng of RNA was reverse transcribed using the High Capacity cDNA Reverse Transcription Kit (Thermo Fisher Scientific, Waltham, MA, USA) according to the protocol. Next, qPCR reactions using the AmpliQ 5× HOT EvaGreen^®^ qPCR Mix Plus (ROX) kit (Novazym, Poznan, Poland) and primers specific for the BPV genome [27] were performed following the protocol of the manufacturer. The reactions were amplified in triplicates using Quant Studio 7 Flex (Thermo Fisher Scientific, Waltham, MA, USA), including non-template control and melt curve analysis to check the specificity of the primers used. The obtained results were analyzed with the ΔΔCt method, including reaction efficiency E which was calculated with the use of the standard curve method [28]. *ACTB* and *UBB* genes were chosen as endogenous controls [29] (Table 1). The analysis with NormFinder software [30] was performed to confirm the stability of gene expression in the samples.

Then, the same workflow and kits were used for the quantification of expression levels of microRNA genes. Primers specific for the investigated pri-microRNAs (mir-101-1, mir-200a, mir-10b, and mir-338) were designed using Primer3 Plus software [31] (Table 1) and checked for specificity with an Ensembl BLAST/BLAT search against the EquCab3.0 genome [32,33].

### 2.3. miR-10b Expression Validation

The expression levels of mature miR-10b underwent additional validation with qRT-PCR, since the expression levels of its precursor investigated in Section 2.2 were opposite to the results obtained in the previous study with NGS [13]. To this end, 600ng of total RNA was reverse transcribed with the TaqMan^®^ Advanced miRNA cDNA Synthesis Kit (Thermo Fisher Scientific, Waltham, MA, USA) according to the protocol. TaqMan^®^ Fast Advanced Master Mix (Thermo Fisher Scientific, Waltham, MA, USA) and miRNA Advanced Assays (478494_mir—has-miR-10b-5p; 477892_mir—has-miR-128-3p as the endogenous control; Thermo Fisher Scientific, Waltham, MA, USA) were used to carry out qPCR reactions according to the manufacturer’s instructions. The reactions were run in triplicates on Quant Studio 7 Flex (Thermo Fisher Scientific, Waltham, MA, USA), including the non-template control. The obtained results were analyzed with the ΔΔCt method, including reaction efficiency E which was calculated with the use of the standard curve method [28]. The analysis with NormFinder software [30] was performed to confirm the stability of gene expression in the samples.

### 2.4. Methylation Analysis of miRNA Localized CpGs

**Figure 1:**
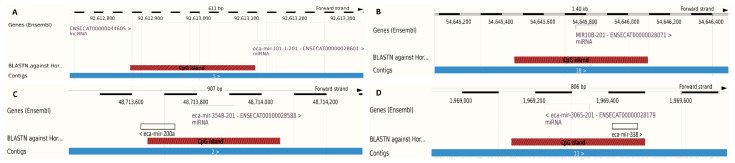
Graphical visualization of CpGs islands identified within the investigated microRNA genes. (**A**)—eca-mir-101-1; (**B**)—eca-mir-10b; (**C**)—eca-mir-200a; (**D**)—eca-mir-338. Blue bars indicate chromosomes; brown bars stand for identified CpG islands; transparent bars indicate microRNA precursors. The graphics were prepared using Ensembl genome browser [33] and EcuCab3.0 genome assembly. The localization of eca-mir-200a and eca-mir-338 was manually added since it is not marked in Ensembl.

The Methyl Primer Express^®^ Software v1.0 program (Applied Biosystems Software; Thermo Fisher Scientific, Waltham, MA, USA) was used to design primers flanking CpG islands localized within the investigated microRNA genes (Figure 1). The amplification reactions were performed by applying the BSP method and HotStartTaq^®^ polymerase (Qiagen, Hilden, Germany). The obtained products were evaluated on 2% agarose gel and then purified using FastAP ™ Thermosensitive Alkaline Phosphatase and *E. coli* exonuclease I (Exo I, Thermo Fisher Scientific, Waltham, MA, USA), according to the manufacturer’s protocol. Sequencing was performed using the BigDye^®^ Terminator v1.1 Cycle Sequencing Kit (Thermo Fisher Scientific, Waltham, MA, USA) and primers used for the BSP amplification (Table 2) following the manual. Unused reaction substrates were removed with the BigDye XTerminator Purification Kit (Thermo Fisher Scientific, Waltham, MA, USA), according to the standard protocol. The electrophoresis of the products was carried out on a 3500xl Genetic Analyzer (Thermo Fisher Scientific, Waltham, MA, USA), while base-calling was performed using Sequencing Analysis Software v5.2 (Thermo Fisher Scientific, Waltham, MA, USA). The obtained chromatograms were inspected with FinchTV v1.4.0 software (Geospiza, Inc., Seattle, WA, USA) to exclude those of low quality. Next, the qualitative analysis of methylation was performed, including the search for methylated cytosins. The quantitative methylation analysis was carried out by applying the Mquant method [34].

### 2.5. Statistics

Data from qPCR and methylation analysis were checked for normality using the Shapiro–Wilk test. Statistical significance testing was performed using the non-parametric Mann–Whitney–Wilcoxon test at the 0.05 significance level. All the tests were run with R package [35].

## 3. Results

The qPCR analysis of the control skin samples with primers specific for BPV showed no relative expression, excluding ongoing BPV infection in these control samples.

In the next stage, we determined the expression levels of the investigated microRNA genes in the sarcoid and control samples. As a result, the decreased expression of all analyzed sequences, namely mir-101-1, mir-10b, mir-200a, and mir-338, was identified in the sarcoid samples compared to the control tissue. The results were statistically significant at the level of 0.05, except for mir-338 (*p* value = 0.211) (Table 3).

The BSP products obtained from the analyzed microRNA genes were subjected to Sanger sequencing. The first stage of the analysis included the qualitative assessment of DNA methylation patterns, based on sequence chromatograms. The presence of cytosines, thymines, or the C/T epigenotype in the bisulfite-converted sequences was the basis for the identification of differences in the level of DNA methylation between the sarcoid tissue samples and the control ones. This part of the analysis showed that all the examined sequences were successfully subjected to bisulfite conversion and confirmed the presence of DNA methylation in them.

**Figure 2:**
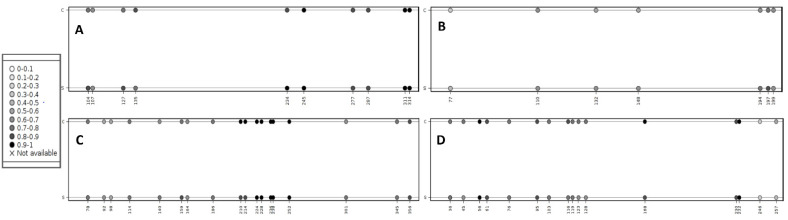
The lollipop-like plot depicting methylation levels of single CpG positions. Figure (**A**) stands for mir-101-1 results; (**B**)—mir-10b results; (**C**)—mir-200a results; (**D**)—mir-338 results. Methylation levels for the control samples are in the upper part of each figure (indicated by letter “C”), while the methylation levels of the sarcoid samples are in the lower part of each figure (indicated by letter “S”). Methylation levels may range between 0 (no methylation, indicated by white circles) and 1 (100% methylation, indicated by black circles). Intermediate values are visualized with grayscale colored circles with 0.1 intervals (the legend). CpG positions are marked on the lower axis. The plot was created with the Methylation plotter web tool [36].

The quantitative analysis using the Mquant method revealed a high average level of CpG island methylation in all tested microRNA genes, both in the sarcoid and control samples (58.8–85.4%). The observed differences in the methylation levels between the sarcoid and control samples were in most cases small, ranging from 1.6% (mir-200a) to 3.4% (mir-338). The exception was mir-10b, for which the mean methylation level in the sarcoid samples was 9% higher than in the control samples. The results were statistically significant at the level of 0.05, except for mir-338 (*p* value = 0.623) (Table 3, Supplementary Files S1–S4). The average methylation levels for the control group and the sarcoid samples for individual CpG positions within the investigated microRNA genes are visualized in Figure 2.

The comparative analysis of the expression quantification and methylation results showed that the direction of the expression changes in the sarcoid samples (downregulation) is opposite to the direction of the methylation level changes (increased levels) for all the investigated miRNA genes (mir-101-1, mir-10b, and mir-200a), except for mir-338. In the case of mir-338, its expression level was decreased in the sarcoid samples and the methylation levels were also lower in the sarcoid samples than in the control ones. However, the obtained results were not statistically significant (Table 3).

Since the expression levels of the miR-10b precursor were opposite to mature miR-10b expression levels revealed in the previous study with NGS [13], we decided to carry out an additional validation of mature miR-10b expression levels with qRT-PCR. The analysis confirmed the previously obtained results [13], that is, the overexpression of miR-10b in sarcoid samples in comparison to the control ones (FC 2.75; *p* value = 0.0005).

## 4. Discussion

We previously showed that miR-101-3p, mir-200a, mir-10b-5p, and mir-338-3p are aberrantly expressed in equine sarcoid tissue [13]. They were documented to take part in biological processes important for the cancerogenesis of different human tumors [11,37,38,39] which, along with our results, implies they may also play important roles in the development of equine sarcoids. Moreover, our in silico analysis revealed the presence of CpG islands within the gene bodies of genes encoding these miRNAs. Therefore, in this study, we assessed the methylation levels of the four miRNA genes in sarcoid samples to elucidate if gene body DNA methylation may be associated with observed changes in the expression levels of these miRNAs during sarcoid neoplastic transformation. Bisulfite sequencing showed that the investigated miRNA genes were highly methylated in both sarcoid tumor samples and control samples. However, mir-101-1, mir-10b, and mir-200a had significantly higher levels of methylation in the sarcoid samples than in the control ones (Table 3). Genomic hypermethylation in cancer is usually identified in CpG islands localized in gene regions [40]. Moreover, Arechederra and colleagues [16] showed that CpG hypermethylation is important for cell tumorigenic properties in liver cancer.

There are no available data on the alterations of DNA methylation levels of mir-101-1, miR-10b, and miR-200a sequences in sarcoids or other equine tumors. The analysis of human gastric cancer [41] and cervical cancer [42] revealed increased methylation levels of the miR-10b promoter in tumor samples. The methylation analysis performed in this study showed increased methylation levels in miR-10b-localized CpG islands. Increased methylation in promoter regions is reported to reduce gene expression levels [14] and similar effects are observed for gene body CpGs. On the other hand, the positive correlation between hypermethylation of intragenic CpGs and gene overexpression has also been observed. Arechederra and colleagues suggested that such an effect is mostly related to genes with oncogenic functions [16]. miR-10b has been shown to act mainly as an oncomiR [43], and in our previous study, we revealed increased expression levels of miR-10b in sarcoid samples [13], which was additionally confirmed by qRT-PCR in this study. It suggests that miR-10b may also be an oncomiR in this type of tumor, which stays in agreement with the positive correlation between methylation and expression levels proposed by Arechederra and colleagues [16]. On the other hand, in this study, we also performed an analysis of expression levels of primary miRNAs, which are the direct products of miRNA gene transcription. For pri-mir-10b, we observed the downregulation of its expression in the sarcoid samples with reference to the control samples, which is opposite to the mature miR-10b expression levels. Although it may seem unusual that miRNA and its precursor expression levels are opposite, it has already been reported at a larger scale [44]. It may stem from the fact that miRNA biogenesis is a multistep process, which may be regulated at each stage and influenced by complex factors, such as, e.g., hairpin precursor stability affected by an SNP (Single-Nucleotide Polymorphism) [45,46]. Moreover, the opposite pri-mir-10b expression and methylation levels stay in agreement with the aforementioned canonical negative influence of DNA methylation on gene expression [14,16].

When it comes to mir-200a, we revealed its increased methylation in the sarcoid samples. Davalos and colleagues [47] showed that it is also hypermethylated in mesenchymal cancer cells. This stays in agreement with the origins of equine sarcoids, which are composed of neoplastically transformed fibroblasts. Furthermore, both pri-mir-200a (this study) and miR-200a [13] were downregulated in the sarcoid samples, which, along with the hypermethylation of their CpG islands, fits into the canonical negative relation between DNA methylation and gene expression [14,16]. A similar effect of increased methylation on lowered gene expression was also observed in non-small-cell lung cancer for miR-200c belonging to the same family as miR-200a [48].

We also showed increased methylation and lowered expression levels of pri-mir-101-1 in the sarcoid samples. The downregulation of mature miR-101, a tumor suppressor, is frequently reported in different human cancers [49]. It is probable that the lowered expression of this miRNA leads to the overexpression of the enhancer of zeste homolog 2 (*EZH2*) and DNA methyltransferase 3A (*DNMT3A*), which belong to the histone methyltransferase family, and finally enhances the DNA methylation of genes [50,51]. As mentioned before, the hypermethylation of genes is often reported in cancer and is of functional importance [16,40]. Thus, the downregulation of miR-101 expression may be part of the mechanism responsible for the dysregulation of epigenetic patterns in tumors, and also in equine sarcoids. miR-101 may play an especially important role since it is engaged in a reciprocal negative feedback loop with *EZH2*, which means that it not only represses *EZH2* translation but is also negatively regulated by *EZH2* itself [52].

The last miRNA analyzed in this study, namely miR-338, is considered to play mainly tumor suppressor roles in different types of cancer. It is established that it is regulated by different types of sequences embracing i.a. circular RNAs and long non-coding RNAs [38]. The analysis of pri-mir-338 expression levels performed in this study showed its downregulation in the sarcoid samples accompanied by decreased levels of DNA methylation. This may imply that its suppressive influence is also present in equine sarcoids. However, the obtained results were not statistically significant, revealing the lack of influence of DNA methylation on pri-mir-338 expression levels in the sarcoid samples.

It should also be emphasized that differences in the methylation levels revealed in this study are not very high (below 10%), which suggests that other mechanisms may also be engaged in the downregulation of the expression of the examined primary miRNAs in equine sarcoids. The heterogeneity of the inspected tissue-samples may also be another reason for this. Moreover, in the case of miR-10b, for which we observed lowered expression levels of its precursor and increased expression levels of the mature sequence, additional mechanisms responsible for the regulation of mature miRNA generation from precursors should be taken into account.

In conclusion, altered methylation profiles of miRNA gene bodies may be responsible for changes in miR-101, miR-10b, and miR-200a expression levels in equine sarcoids. However, they seem to be a part of the complex regulatory networks leading to the dysregulation of miRNA expression at different levels of genome organization and functioning. Their in-depth elucidation requires further studies.

## Figures and Tables

**Table 1:** qPCR primer sequences used in the study.

Gene Name	Forward Sequence (5′–3′)	Reverse Sequence (5′–3′)	Product Size (bp)
BPV	TGCAGTTGTCTTTGCAGGAG	AGCACCGTTTAGGTTCTGACAT	104
*ACTB*	CCAGCACGATGAAGATCAAG	GTGGACAATGAGGCCAGAAT	88
*UBB*	GCAAGACCATCACCCTGGA	CTAACAGCCACCCCTGAGAC	206
eca-mir-101-1	TCACAGTGCTGATGCTGTCA	TAGGGGAGGCACAATATGGA	178
eca-mir-200a	CTTACCGGACAGTGCTGGAT	CCGATGTGGCTGAACTGAC	169
eca-mir-10b	ATTGCCACCAAGTCCTTCAG	TGAAGTTTTTGCATCGACCA	237
eca-mir-338	CGGAAGAAATGGTGATGGAC	AGCTGCCCTCTTCAACAAAA	132

**Table 2:** BSP primer sequences used in the study.

Gene Name	Forward Sequence (5′–3′)	Reverse Sequence (5′–3′)	Product Size (bp)	Number of Analyzed CpG Site
eca-mir-101-1	GAGGTTAGGGAGATAGTAAGTTTAGG	ACCTTTAAAACTAACAACATCAACA	384	10
eca-mir-200a	TTATTTTGGAGAGAGTAGGGG	CCTAACCCTAATAATCTATCCCA	419	18
eca-mir-10b	GGTTGGTAGTAGTTTGGGTATTTG	CCAAAATCTAACCCTTTAACCC	367	7
eca-mir-338	GAGGGATGGTTTTGTTTTG	TACATCTACCACACAACTACTATACCA	314	14

**Table 3:** Data on relative expression and DNA methylation levels of the investigated miRNA genes in the sarcoid tissue and control samples. Fold change expressed as −1/FC.

	mir-101-1	mir-10b	mir-200a	mir-338
Relative average expression level in the sarcoid samples	0.14	0.12	0.18	0.21
Relative average expression level in the control samples	0.27	0.27	0.50	0.50
**Fold change**	**−1.92** **(*p* value = 0.031)**	**−2.27** **(*p* value = 0.050)**	**−2.78** **(*p* value = 0.004)**	**−2.38** **(*p* value = 0.211)**
Average CpG methylation level in the sarcoid samples	85.4%	67.8%	79.9%	73.9%
Average CpG methylation level in the control samples	82.5%	58.8%	78.3%	77.3%
**Methylation difference**	**2.9%** **(*p* value = 0.009)**	**9.0%** **(*p* value = 0.011)**	**1.6%** **(*p* value = 1.49 × 10^−5^)**	**−3.4%** **(*p* value = 0.623)**

## Supplementary Materials

The following supporting information can be downloaded at: https://www.mdpi.com/article/10.3390/cells11121917/s1, Supplementary File S1. A fragment of the chromatogram of the mir-101-1 region indicating the differences in the CpG methylation pattern between the sarcoid and control samples (A—control sample, B—sarcoid; black arrows show the analyzed CpG sites). Supplementary File S2. A fragment of the chromatogram of the mir-10b region indicating the differences in the CpG methylation pattern between the sarcoid and control samples (A—control sample, B—sarcoid; black arrows show the analyzed CpG sites). Supplementary File S3. A fragment of the chromatogram of the mir-200a region indicating the differences in the CpG methylation pattern between the sarcoid and control samples (A—control sample, B—sarcoid; black arrows show the analyzed CpG sites). Supplementary File S4. A fragment of the chromatogram of the mir-338 region indicating the differences in the CpG methylation pattern between the sarcoid and control samples (A—control sample, B—sarcoid; black arrows show the analyzed CpG sites).
