# Supplementary figures and images for: Methylation Status of Gene Bodies of Selected microRNA Genes Associated with Neoplastic Transformation in Equine Sarcoids
Source: Cells. 2022 Jun 14;11(12):1917. doi: 10.3390/cells11121917 (PMC9221590; doi:10.3390/cells11121917)

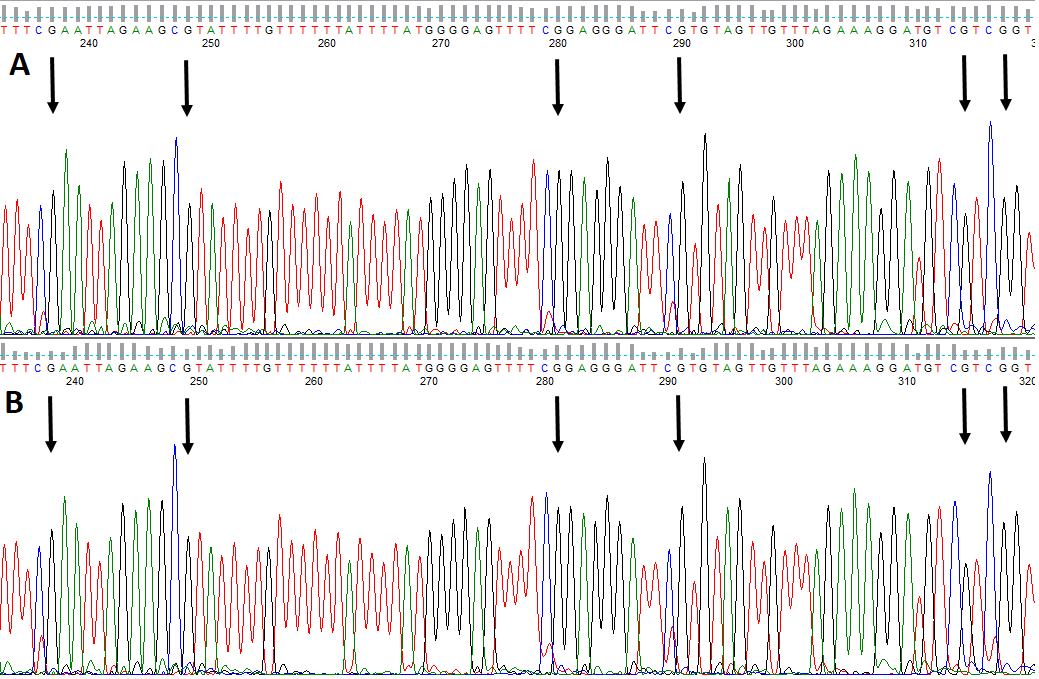

Supplement: Supplementary file 1 [file cells-11-01917-s001.zip › cells-1743553-supplementary/Supplementary file S1.png]

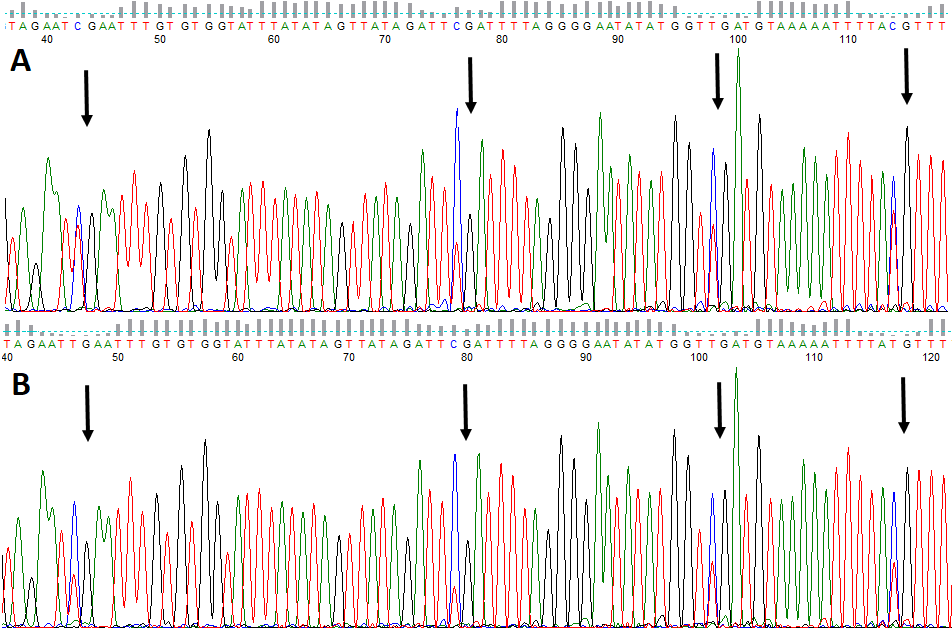

Supplement: Supplementary file 1 [file cells-11-01917-s001.zip › cells-1743553-supplementary/Supplementary file S2.png]

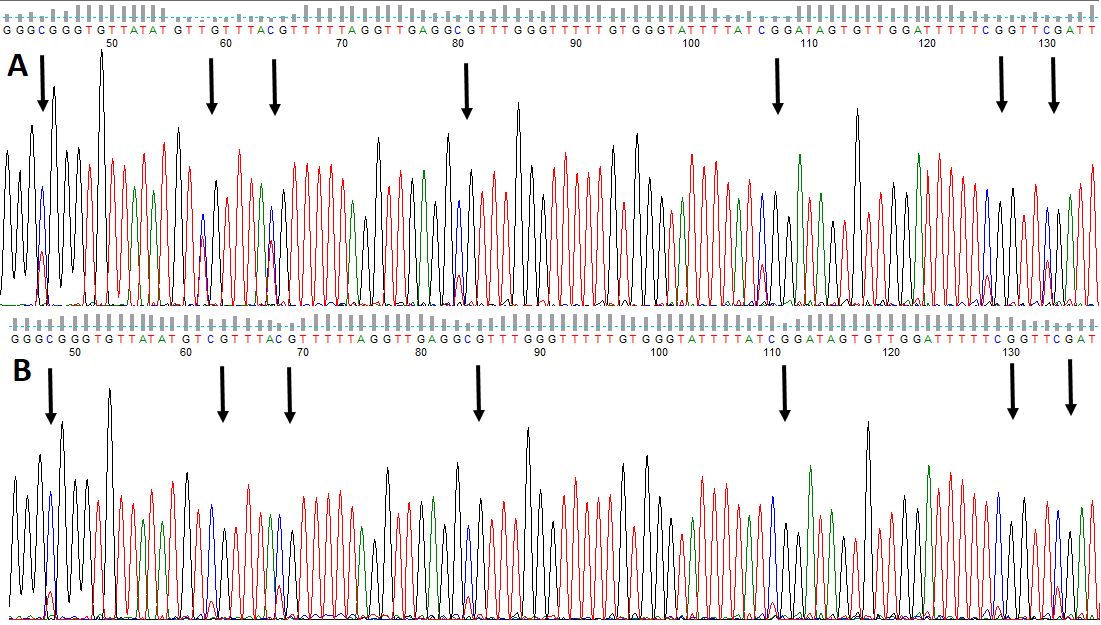

Supplement: Supplementary file 1 [file cells-11-01917-s001.zip › cells-1743553-supplementary/Supplementary file S3.png]

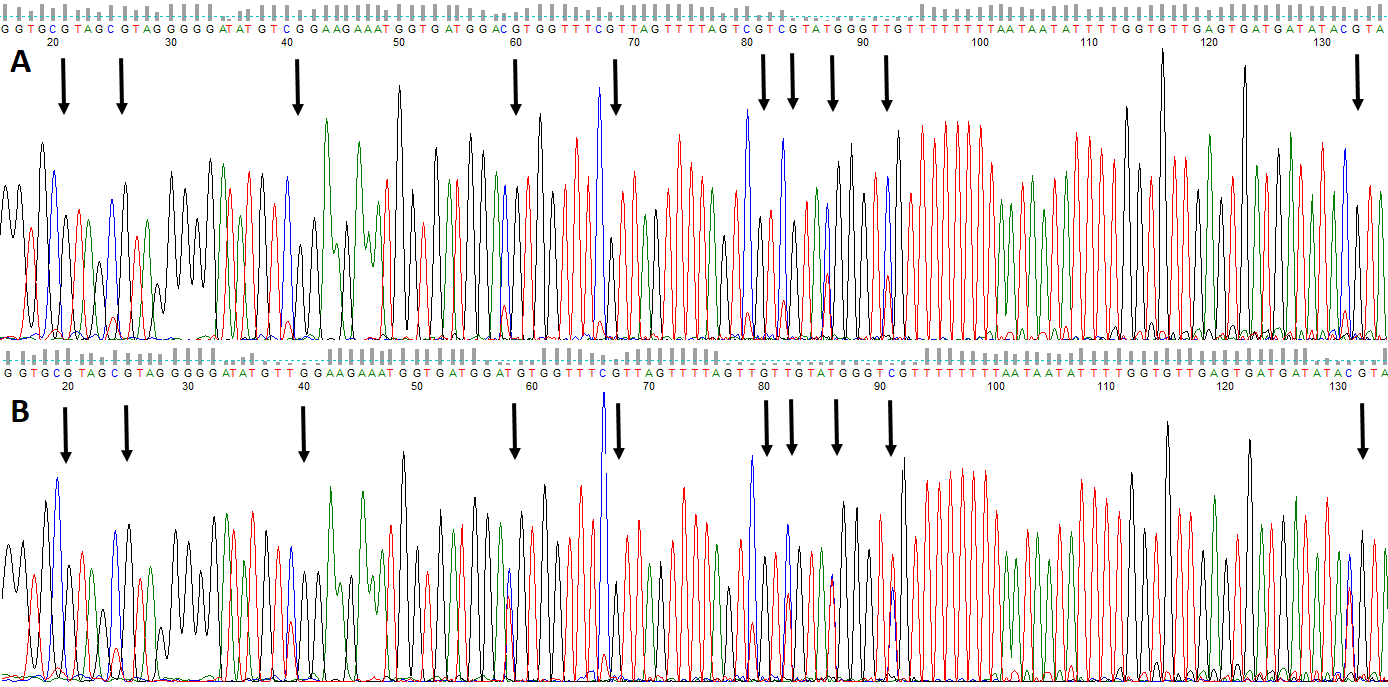

Supplement: Supplementary file 1 [file cells-11-01917-s001.zip › cells-1743553-supplementary/Supplementary file S4.png]
